# Psychiatric symptoms and the association with parents’ psychiatric symptoms among recently arrived asylum-seeking children in Finland

**DOI:** 10.1007/s10578-022-01371-2

**Published:** 2022-05-19

**Authors:** Heidi Parviainen, Olli Kiviruusu, Riikka Lämsä, Natalia Skogberg, Anu E Castaneda, Päivi Santalahti

**Affiliations:** 1 Department of Public Health, University of Turku, FI-20014 Turku, Finland; 2 Department of Public Health and Welfare, Finnish Institute for Health and Welfare (THL), Helsinki, Finland; 3 Department of Public Health, University of Helsinki, Helsinki, Finland; 4 Department of Child Psychiatry, University of Turku, Turku, Finland

**Keywords:** Asylum-seeking children, Accompanied refugee children, Psychiatric symptoms, Parent psychopathology, Mental Health

## Abstract

This study examined psychiatric symptoms and the association with parents’ psychiatric symptoms among recently arrived accompanied asylum-seeking children in the age groups of 2–6 years (n = 93) and 7–12 years (n = 91). Children and parents were assessed using mental health and trauma measures (SDQ, HSCL-25 and PROTECT). The prevalence of total difficulties was 34.9% among 2–6-year-olds and 29.6% among 7–12-year-olds. The most common symptoms in both age groups were peer problems, followed by conduct problems among 2–6-year-olds and emotional symptoms among 7–12-year-olds. In both age groups, the children’s emotional symptoms were associated with the parents’ anxiety and depression as well as the trauma symptoms, while the conduct problems were only associated with the parents’ trauma symptoms. In conclusion, peer problems as well as conduct problems and emotional symptoms are common among recently arrived asylum-seeking children. To support the mental health of these children, both children and parents need adequate support.

**Supplementary Information:**

The online version contains supplementary material available at 10.1007/s10578-022-01371-2.

## Introduction

In 2019, European countries received over 670 000 new asylum seekers and nearly a third of them were children [1]. Asylum-seeking and refugee children have a higher prevalence of psychiatric symptoms compared to non-refugee populations [2, 3]. They have high rates of trauma symptoms, depression, and anxiety, as well as heightened levels of externalising problem behaviours such as aggression and hyperactivity [2, 4]. The majority of previous studies exploring the mental health of asylum-seeking and refugee children have focused on vulnerable unaccompanied adolescents (migrating without a parent or guardian), and when accompanied children have been addressed, the children have been mainly school-aged, as revealed by two recent reviews [5, 6]. There has been much less discussion on the mental health of accompanied children of which over 90% are asylum-seeking children and primarily younger than unaccompanied children, and especially the mental health of under school-aged children has been neglected [1, 6]. In 2016, Van Os and colleagues [7] presented a systematic review on existing knowledge of recently arrived refugee children in the host country. The review presented six studies on mental health symptoms of accompanied children and of these only two addressed specifically under school-aged children [7]. While the reviewed studies reported high levels of traumatic stress or emotional symptoms [8, 9] or PTSD (post-traumatic stress disorder) [8, 10], there seem to be – based on the review – also evident gaps in the literature, as the studies of accompanied children focused typically on certain ethnic groups and even the most recent study was published two decades ago. A more recent nationwide register-based study presenting the health status of newly arrived asylum-seeking minors in Denmark found that more than 40% of the 0–17-year-old children were in need of psychosocial support and further assessment; however, the symptoms of young children or accompanied children were not separately addressed [11].

The age of the children as well as the phase of the asylum-seeking journey and its duration influence exhibition of psychiatric symptoms among asylum-seeking children. Hence, it is crucial to understand the mental health of these children prior to the influence of the factors related to the stay in the country of asylum. Emotional and trauma symptoms have higher rates of occurrence in children who have arrived recently than among children who have been displaced for a long period of time, whereas hyperactivity has been shown to be higher among those displaced for more than 2 years [2]. Moreover, asylum-seeking children enter the destination country at different ages. Usually, studies among refugee children have found an increasing prevalence of mental health problems with age [3]. This is associated with older children experiencing more and more severe traumatic events as they have a greater cognitive capacity to process their impact [12, 13].

Parents’ mental health has a significant impact on the mental health of children [14]. The mechanisms of the transgenerational risk of mental illness can be categorized into genetic risk transmission, prenatal influences, parent-child interactions, family processes and conditions, and social influences from outside the family [15]. The socioecological contexts of asylum-seeking children and their families are often characterized by trauma, loss and cumulative stressors, and studies have shown that immigrant status affects the mechanisms of the transgenerational risk [16–18]. There is a strong association between the mental health problems of refugee parents and children, and good parental mental health is an important protective factor [19]. However, the capacity of a refugee parent to meet the child’s changing needs can be compromised as a result of the parent’s own trauma, cultural dislocation, loss of support networks and non-supportive living conditions [20, 21]. Consequently, it is important to study the associations between recently arrived accompanied asylum-seeking children’s and parents’ psychiatric symptoms, prior to the influence of post arrival factors.

Thus, there is a significant need for research on mental health of accompanied asylum-seeking children at the time of arrival especially in the younger age groups. This information on mental health is essential for finding out how well-being can best be promoted post-arrival [22]. Most previous studies are limited to certain ethnic groups, have some methodological weaknesses or are rather old [7, 11]. To address the gaps in the literature, this study aims to examine psychiatric symptoms of recently arrived accompanied asylum-seeking children in the age groups of 2–6 years and 7–12 years. Additionally, the aim is to explore whether the child’s symptoms are related to the parents’ anxiety and depression as well as trauma symptoms.

### Methods

#### Participants and procedure

The data of the present study were derived from the Asylum Seekers Health and Wellbeing (TERTTU) Survey carried out by the Finnish Institute for Health and Welfare (THL) in collaboration with the Finnish Immigration Service. The study protocol and the description of the data collection methodology have been described in more detail elsewhere [23, 24]. Briefly, a sample of all first-time asylum applicants during the period of 19.2.2018 and 30.11.2018 was drawn from the Finnish Immigration Service’s electronic asylum database. All asylum applicants had submitted an application for international protection to Finland in fear of persecution, war or other types of insecurity. Due to logistical reasons, the sample was not drawn during two short periods (30.4.–13.5.2018; 27.8.–9.9.2018), leading to exclusion of 158 first-time applicants to Finland. Additionally, 63 first-time applicants were excluded during the summer period of 2018 due to an IT error. Following these exclusions, the final sample of the TERTTU Survey consisted of 1433 first-time applicants (n = 992 adults, n = 365 accompanied children and n = 76 unaccompanied children).

Participants were recruited approximately two weeks following arrival in Finland. The majority (79%) of participants took part in the study within 30 days and 14% between 31 and 60 days of arrival in Finland. Data collection was carried out by multilingual research assistants who spoke Somali, Arabic, Dari, Persian, Kurdish/Sorani, Portuguese, French or Russian, in addition to Finnish and English. The multilingual research assistants received a week and a half long training for the administration of the survey, and during the survey the fieldwork manual and daily contact with the fieldwork coordinator was in use. There were altogether seven assistants, and all except one had educational background in healthcare. All the study material was translated into Somali, Arabic, Persian, Kurdish/Sorani, Russian and English. Measures used in the study were readily available in these languages for use from those who designed the original questionnaires, and from previous studies that have been conducted in these languages among migrant populations. Additionally, the information letter and consent form were translated into Turkish. Majority of interviews (62%) were conducted directly by multilingual research assistants. In the rest of the cases, a professional interpreter was used. Altogether, data collection was carried out in 31 languages.

The study material, including the information letter, consent forms and interviews, were compiled for each of the four age groups: under school-aged children (0–6 years); school-aged children (7–12 years); adolescents (13–17 years) and adults (18 years and older) [23, 24]. Data collection consisted of a standardised health examination and a face-to-face-interview during which some of the data (reproductive health and mental health symptoms) were gathered with a self-administered questionnaire. Trauma-related symptoms were interviewed, whereas data on mental health symptoms was primarily collected with self-administered questionnaires. In cases when the participant was illiterate, had difficulties completing the questionnaire themselves, or the questionnaire was not available in the language spoken by the participant, they were interviewed by the research nurse. In cases when the language spoken by the participant was other than those spoken by the multilingual research assistants, professional interpreters from accredited companies were used to facilitate administration of the questionnaires. Exactly the same questionnaires were used whether self-reported or interviewed. The study also included questions on living conditions, health, physical and social functional capacity, quality of life, experiences of potentially traumatic events, experienced of violence, life-style habits and service use. Interviews of children also included questions on early childhood and development. The guardians of accompanied children under the age of 13 years answered the interview and the questionnaire on the behalf of the child. In such cases, the child participated him/herself in the health examination only. Children aged 13 years and older as well as adults took part in all parts of the study by themselves. Ethical approval was granted by the Coordinating Ethics Committee of the Helsinki and Uusimaa Hospital District (HUS/3330/2017). All the adult participants provided written informed consent. The guardians of children and adolescents aged 17 years and younger provided written informed consent for the child’s participation in the study. Additionally, children aged seven years and older provided their own informed written consent.

The present study focuses on a sub-sample of accompanied children aged 2–12 years (n = 184) and their parents (n = 178, consisting of n = 110 mothers and n = 68 fathers). In this study, the term ‘parent’ refers to both parents and guardians. The participation rate for adults was 79% and for accompanied children 78% [24]. Of the children included in this study, 93 (50.5%) were aged 2–6 years and 91 (49.5%) were aged 7–12 years. In total, 119 families were included, out of which 78 had 2–6-year-old children and 71 families had 7–12-year-old children. Of the 119 families included in the study, in 59 families both parents took part in the study, in 29 families both parents were in Finland but only one parent took part in the study, and in 31 families there was only one parent, a mother. In the latter group consisting of mothers who came alone with their children, fathers were reported to be deceased, to reside in another country, to be missing or not to exist. Out of all the 119 families that participated, 46 families had one child, 46 had two children, 20 had three children and 7 had more than three children, based on the number of children participating in the TERTTU Survey. In total there were 51 families that had more than one child included in the study sample.

## Measures

### Demographic details

In this study, the sociodemographic factors used in the analysis were the child’s gender, the parent’s education level and family type. The parent’s education level was composed of eight categories: not known, none, basic education three years or less, basic education 4–6 years, basic education 7–9 years, secondary school, vocational training and post-secondary. The parent’s education level was divided into two categories: (1) basic education 9 years or less and (2) secondary school, vocational training or higher. It was further organized into a dichotomous variable according to the highest education level of both parents or the only parent in the study. Family type comprised (1) families with two parents, either both in the study or one in the study and the other in Finland; (2) families with one parent, the other not in Finland or not existing.

### Prevalence of psychiatric symptoms in children

The Strengths and Difficulties Questionnaire (SDQ) was used, consisting of 25 items assessing children’s psychiatric symptoms in five subscales: emotional symptoms, conduct problems, hyperactivity, problems with peers, and prosocial behavior [25]. In this analysis, the subscales excluding prosocial behavior were used. SDQ is widely used internationally and has been validated in various research and community settings showing adequate psychometric properties [26–28]. The questionnaire was completed by parents. For 2–4-year-old children, 3 out of 25 statements have different wording compared to the questionnaire for 4–17-year-old children. The British normative scoring bands dividing 80% of children to ‘normal’ category, 10% to ‘borderline’ and 10% to ‘abnormal’ were used, taking into account different cut-off scores for 2–4-year-old children and older children [25, 29]. The borderline and abnormal categories were combined into one category.

### Prevalence of psychiatric symptoms in parents

The anxiety and depression symptoms of parents were assessed with the Hopkins Symptom Checklist-25 (HSCL-25). It has been widely used in refugee populations to assess symptoms of anxiety and depression [30, 31]. The total score is the average of all 25 items. The cut-off score of 1.75 was used for the clinical range [32]. The parents’ anxiety and depression symptoms were organized into dichotomous variable of both parents or the only parent in the study with anxiety and depression in the clinical range (yes/no), based on the known presence of at least one healthy parent in the family buffering the child from the effects of mental ill health [33, 34].

The parents were interviewed about trauma symptoms using the Process of Recognition and Orientation of Torture Victims in European Countries to Facilitate Care and Treatment (PROTECT) questionnaire [35]. It evaluates the likelihood of traumatic experiences based on trauma symptoms. The answers are categorized into three categories: low risk, medium risk and high risk. Medium and high risk were combined into one category. For the purposes of this study, the outcome of PROTECT questionnaire is referred to as ‘trauma symptoms’ as it covers the most significant symptoms of psychological trauma [35]. The parents’ trauma symptoms were organized into dichotomous variable of both parents or the only parent in the study with trauma symptoms in medium or high risk category (yes/no), based on the known presence of at least one healthy parent in the family buffering the child from the effects of mental ill health [33, 34].

### Statistical analysis

Sociodemographic factors and the children’s psychiatric symptoms were reported in frequencies separately for the two age groups.

Generalized linear mixed models were used to analyze associations of the parents’ psychiatric symptoms with the four SDQ subscales and total difficulties in the two age groups. Mixed modelling accounts for nested sources of variability, which means involving units at a lower level (i.e., individuals) nested within units at a higher level (i.e., families). Analyses were conducted first with only the parents’ anxiety and depression or the parents’ trauma symptoms as the independent variable (univariate) and this was followed by models adjusting for sociodemographic factors (multivariate). The sociodemographic factors used were the child’s gender, the parent’s education level and family type. The associations of parents’ psychiatric symptoms and SDQ subscales and total difficulties have been presented as odds ratios and 95% confidence intervals obtained from generalized linear mixed models.

The level for statistical significance was set at *p* < 0.05. The statistical analyses were performed with SPSS software version 27.

## Results

### Participants

The sociodemographic characteristics of the study participants are presented in Table 1. Among both 2–6-year-old and 7–12-year-old children, over 70% of mothers (79.5% and 74.2%) and about 90% of fathers (91.1% and 89.2%) had an education level of at least secondary education. In both age groups of children, over one third of the mothers (36.6% and 38.5%) and over one fifth of the fathers (21.4% and 22.2%) reported anxiety and depression symptoms within the clinical range. Trauma symptoms were even more prominent, with over 40% of mothers (44.4% and 53.8%) and fathers (40.0% and 40.5%) reporting medium or high-risk trauma symptoms. Among 2–6-year-old children, the most frequent region of origin was North Africa and Middle East (57.0%), followed by Russia and Former Soviet Union (30.1%). Among 7–12-year-old children, the most frequent region of origin was Russia and Former Soviet Union (40.7%), followed by North Africa and Middle East (35.2%). The sample characteristics are presented in more detail in Online Resource 1.

**Table 1:** Sociodemographic characteristics of the participants and parents’ psychiatric symptoms

*Child*		Child, 2–6 years *n* = 93 *n* (%)	Child, 7–12 years *n* = 91^a^ *n* (%)
Gender	Male	43 (46.2)	51 (56.7)
	Female	50 (53.8)	39 (43.3)
***Mother***		***n*** **= 73** ^**a**^ ***n*** **(%)**	***n*** **= 66** ^**a**^ ***n*** **(%)**
Education level	Basic education 9 years or less	15 (20.5)	17 (25.8)
	Secondary, vocational or higher	58 (79.5)	49 (74.2)
Anxiety and depression at clinical range^b^		26 (36.6)	25 (38.5)
Trauma symptoms in medium or high-risk category^c^		32 (44.4)	35 (53.8)
***Father***		***n*** **= 45** ^**a**^ ***n*** **(%)**	***n*** **= 37** ^**a**^ ***n*** **(%)**
Education level	Basic education 9 years or less	4 (8.9)	4 (10.8)
	Secondary, vocational or higher	41 (91.1)	33 (89.2)
Anxiety and depression in the clinical range^b^		9 (21.4)	8 (22.2)
Trauma symptoms in medium or high-risk category^c^		18 (40.0)	15 (40.5)
***Family***		***n*** **= 78** ^**a**^ ***n*** **(%)**	***n*** **= 71** ^**a**^ ***n*** **(%)**
Family type	Two parents, either both in the study or one in the study and the other in Finland	60 (76.9)	50 (70.4)
	One parent, the other not in Finland or not existing	18 (23.1)	21 (29.6)
Both parents or the only parent in the study with anxiety and depression in the clinical range^b^		19 (25.7)	20 (29.0)
Both parents or the only parent in the study with trauma symptoms in medium or high-risk category^c^		30 (39.0)	29 (41.4)

^a^ *n* varies slightly (max n-4) according to measure due to missing information.

^b^Hopkins Symptom Checklist-25 (HSCL-25)

^c^Process of Recognition and Orientation of Torture Victims in European Countries to Facilitate Care and Treatment (PROTECT)

## Prevalence of psychiatric symptoms in children

The prevalence of psychiatric symptoms based on SDQ by age group and gender are presented in Table 2. The prevalence of SDQ total difficulties was 34.9% among 2–6-year-old children and 29.6% among 7–12-year-old children. The most common psychiatric symptoms among 2–6-year-old children were peer problems (40.7%) and conduct problems (38.4%). Among 7–12-year-old children, the most common psychiatric symptoms were peer problems (40.7%) and emotional symptoms (39.5%). Hyperactivity was the least common symptom in both groups (25.6% and 16.0%). Regarding conduct problems of 2–6-year-old children, over half of the males had symptoms above the normal score (51.3%), and these symptoms were the only ones in the analyses with significant gender difference (females 27.7%).

**Table 2:** Prevalence of psychiatric symptoms based on SDQ among asylum-seeking children by age group and gender

	Child, 2–6 years % (95% CI)				Child, 7–12 years % (95% CI)			
	All *n =* 86	Male *n =* 39	Female *n =* 47	p*	All *n =* 81	Male *n =* 44	Female *n =* 37	p*
Total difficulties	34.9 (24.4–44.8)	38.5 (23.1–56.2)	31.9 (17.9–45.8)	0.526	29.6 (19.5–39.7)	31.8 (18.9–46.9)	27.0 (12.8–41.9)	0.638
Emotional symptoms	33.7 (23.8–43.5)	30.8 (17.1–46.9)	36.2 (22.9–50.0)	0.598	39.5 (28.6–50.0)	36.4 (22.4–51.1)	43.2 (26.7–60.7)	0.528
Conduct problems	38.4 (28.0-48.8)	51.3 (34.4–66.7)	27.7 (14.6–41.2)	0.025	27.2 (17.1–37.7)	25.0 (13.2–38.5)	29.7 (14.3–46.1)	0.634
Hyperactivity	25.6 (15.9–34.5)	33.3 (20.0-48.7)	19.1 (8.2–31.6)	0.133	16.0 (8.3–25.0)	15.9 (5.6–27.7)	16.2 (5.3–29.0)	0.970
Peer problems	40.7 (30.5–50.6)	43.6 (28.2–59.4)	38.3 (23.8–52.3)	0.619	40.7 (30.1–51.2)	43.2 (29.2–58.5)	37.8 (23.1–53.8)	0.626

*p-value for the difference between males and females, Chi square

## The association between the psychiatric symptoms of children and parents

Results from the generalized linear mixed models analyzing the association between the children’s psychiatric symptoms and parents’ anxiety and depression, as well as parents’ trauma symptoms, are presented in Table 3. In the unadjusted model for 2–6-year-old children, the parents’ anxiety and depression were associated with the child’s SDQ total difficulties (*p* = 0.017), emotional symptoms (*p* = 0.002), conduct problems (*p* = 0.042) and peer problems (*p* = 0.019), and parents’ trauma symptoms were associated with child’s SDQ total difficulties (*p* = 0.038), emotional symptoms (*p* = 0.002) and conduct problems (*p* = 0.011). These associations remained significant in the adjusted models with the exception of the parents’ anxiety and depression and the child’s conduct problems (*p* = 0.061). In the unadjusted model for 7–12-year-old children, the parents’ anxiety and depression were associated with the child’s emotional symptoms (*p* = 0.001) and the parents’ trauma symptoms with the child’s emotional symptoms (*p* = 0.006), conduct problems (*p* = 0.012) and peer problems (*p* = 0.013). These associations remained significant in the adjusted models.

**Table 3:** Odds ratios (OR) and their 95% confidence intervals (95% CI) for parents' anxiety and depression as well as trauma symptoms predicting the child's psychiatric symptoms based on SDQ.

Fixed effects		Parents' anxiety and depression OR (95% CI)^b^		Parents' trauma symptoms OR^a^ (95% CI)^c^	
		**Unadjusted**	**Adjusted** ^**a**^	**Unadjusted**	**Adjusted** ^**a**^
*Child, 2–6 years*					
	**Total difficulties**	4.32 (1.31–14.27)*	4.04 (1.12–14.57)*	3.09 (1.07–8.96)*	3.40 (1.03–11.17)*
	**Emotional symptoms**	6.12 (1.94–19.26)**	6.11 (1.75–21.32)**	5.39 (1.84–15.78)**	9.70 (2.25–41.79)**
	**Conduct problems**	3.16 (1.05–9.57)*	2.99 (0.86–10.36)	3.62 (1.36–9.67)*	3.59 (1.15–11.19)*
	**Hyperactivity**	1.49 (0.52–4.27)	1.48 (0.41–5.26)	1.84 (0.68–4.98)	1.74 (0.54–5.67)
	**Peer problems**	3.74 (1.25–11.19)*	3.52 (1.04–11.92)*	2.14 (0.83–5.50)	2.04 (0.65–6.40)
*Child, 7–12 years*					
	**Total difficulties**	3.19 (0.89–11.37)	3.07 (0.80-11.83)	3.21 (1.00-10.34)	3.07 (0.75–12.57)
	**Emotional symptoms**	10.21 (2.60-40.09)**	7.89 (2.11–29.54)**	5.40 (1.65–17.65)**	5.17 (1.42–18.73)*
	**Conduct problems**	2.01 (0.64–6.32)	1.85 (0.47–7.33)	4.13 (1.38–12.34)*	4.29 (1.01–18.30)*
	**Hyperactivity**	0.86 (0.20–3.67)	0.95 (0.16–5.79)	1.40 (0.41–4.77)	1.91 (0.40–9.11)
	**Peer problems**	1.76 (0.58–5.29)	1.82 (0.55–6.05)	3.60 (1.31–9.86)*	3.66 (1.06–12.64)*

^a^Adjusted for child's gender, family type and parent's education. Family type: Two parents = 0, one parent = 1. Parent's education: Highest education level of both parents or the only parent in the study 9 years of basic education or less = 1, secondary school, vocational school or higher = 0.

^b^Both parents or the only parent in the study with Hopkins Symptom Checklist-25 (HSCL-25) in the clinical range of 1.75 points or more = 1, else = 0.

^c^Both parents or the only parent in the study with Process of Recognition and Orientation of Torture Victims in European Countries to Facilitate Care and Treatment (PROTECT) in medium or high-risk category = 1, else = 0.

*p < 0.05, **p < 0.01, ***p < 0.001.

The prevalence of psychiatric symptoms based on SDQ by age group and the parents’ psychiatric symptoms is presented in Table 4. In both age groups, children with parents with normal scores in psychiatric symptom measures had less psychiatric symptoms compared with those whose parents had clinically significant scores, but the prevalence of children’s psychiatric symptoms remained high nonetheless.

**Table 4:** Prevalence of psychiatric symptoms based on SDQ among asylum-seeking children by age group and parent's psychiatric symptoms

	Child, 2–6 years % (95% CI)						Child, 7–12 years % (95% CI)						
	**Both parents or the only parent in the study with anxiety and depression** ^**a**^			**Both parents or the only parent in the study with trauma symptoms** ^**b**^			**Both parents or the only parent in the study with anxiety and depression** ^**a**^			**Both parents or the only parent in the study with trauma symptoms** ^**b**^			
	Clinical *n* = 22	Normal *n* = 63	p*	Clinical *n* = 37	Normal *n* = 48	p*	Clinical *n* = 22	Normal *n* = 58	p*	Clinical *n* = 32	Normal *n* = 49	p*	
Total difficulties	59.1 (37.5–80.0)	25.4 (15.0-37.1)	0.004	48.6 (33.3–64.7)	25.0 (13.0-36.7)	0.024	45.5 (23.8–66.7)	22.4 (12.1–33.3)	0.042	43.8 (26.8–60.7)	20.4 (9.6–32.5)	0.025	
Emotional symptoms	63.6 (42.1–83.3)	22.2 (11.9–33.3)	< 0.001	54.1 (37.5–69.2)	18.8 (7.8–29.8)	< 0.001	72.7 (53.8–89.5)	24.1 (13.8–35.7)	< 0.001	59.4 (41.2–76.9)	24.5 (12.8–37.9)	0.002	
Conduct problems	59.1 (36.4–80.0)	31.7 (19.7–43.8)	0.023	54.1 (37.5–70.0)	25.0 (13.6–37.5)	0.006	36.4 (16.7–56.5)	22.4 (11.7–32.8)	0.205	43.8 (25.7–62.5)	16.3 (6.5–28.3)	0.007	
Hyperactivity	31.8 (12.5–51.9)	23.8 (13.7–34.8)	0.460	32.4 (17.4–48.5)	20.8 (9.5–33.3)	0.226	13.6 (0.0-29.6)	15.5 (6.5–25.0)	0.833	18.8 (5.6–33.3)	14.3 (5.3–25.0)	0.593	
Peer problems	63.6 (42.9–83.3)	31.7 (19.4–43.3)	0.009	51.4 (35.9–68.8)	33.3 (19.6–46.9)	0.094	50.0 (29.2–70.8)	36.2 (23.9–48.1)	0.261	59.4 (40.7–76.7)	28.6 (16.3–41.9)	0.006	

*p-value for the difference between clinical and normal range, Chi square

## Discussion

This study examined the psychiatric symptoms of recently arrived accompanied asylum-seeking young children in two age groups and whether these were associated with the parents’ psychiatric symptoms. The results show that accompanied asylum-seeking children have a high prevalence of psychiatric symptoms. Approximately one third of the children in both age groups had SDQ total difficulties above normal score as assessed by parents. The most common symptoms in both age groups were peer problems, followed by conduct problems among 2–6-year-old children and emotional symptoms among 7–12-year-old children. Among the youngest group of children, over half of the males had conduct problems above the normal score. In both age groups, the children’s emotional symptoms were associated with the parents’ anxiety and depression as well as the parents’ trauma symptoms, and the children’s conduct problems were only associated with parents’ trauma symptoms. The children’s peer problems were associated with the parents’ anxiety and depression in the younger age group, and with the parents’ trauma symptoms in the older age group.

The findings regarding high prevalence of psychiatric symptoms in both age groups of asylum-seeking children are in line with previous studies often comprising of school-aged or unaccompanied children [2, 6, 36], as well as recently arrived asylum-seeking children particularly [7, 37]. The parents estimated the prevalence of SDQ total difficulties (34.9% in 2–6-year-olds and 29.6% in 7–12-year-olds) more than twice as high as the worldwide pooled prevalence of mental disorder in children and adolescents (13.4%) [38]. Children are often dependent on other people to recognize their psychiatric problems and need assistance in getting care; therefore, it is crucial that parents recognize their children’s psychiatric symptoms [39]. Our results seem to imply that this is the case given the high SDQ scores reported by parents. Yet, this does not imply that parents also perceive their children’s symptoms as problematic and regard the children in need of mental health care [40]. A culturally sensitive approach is needed when evaluating the psychiatric symptoms of asylum-seeking children and treatment planning, taking into account possible alternative perceptions of the child’s well-being [41].

The children’s emotional problems were associated with the parents’ anxiety and depression as well as the parents’ trauma symptoms in both age groups. According to previous literature, this may be related to lower emotional availability and increased parenting stress of the parents as a result of parental psychopathology, which may be particularly notable regarding asylum-seeking children, who have often lost the support network of a larger family [42, 43]. Both maternal and paternal psychopathology are associated with negative consequences for children’s development, including internalizing problems [44, 45].

The children’s conduct problems were associated with the parents’ trauma symptoms in both age groups, which supports the literature showing that parental trauma increases the children’s behavioural and other mental health problems, even if the child is not exposed to the traumatic event [46, 47]. Previous findings in the literature state that parents’ traumatic experiences contribute to their PTSD, which in turn contributes to harsh parenting and poorer mental health outcomes of refugee children [46]. Among traumatized refugees, family-related anger is a major clinical concern [48]. Parental symptoms of PTSD are suggested to be related to parental insensitivity and the child’s insecure attachment [49]. Harsh parenting and insecure attachment are risk factors for conduct problems [50, 51]. It is also plausible that children have been exposed to the same traumatic events as their traumatized parents, thus explaining the behavioural symptoms. Regarding the trauma symptoms of parents and children, the children’s exposure to parental PTSD could aggravate the effect of previous trauma in children through disruption of adaptive systems, such as family support and secure attachment [19, 47]. Regarding the high prevalence of conduct problems especially in young males, it is critical to note that childhood conduct problems are associated with multiple negative outcomes in adulthood, such as psychiatric and somatic diseases, psychosocial difficulties and unemployment [52]. Thus, the early recognition and intervention is of utmost importance.

With reference to a high prevalence of peer problems at arrival, it is crucial to note that during resettlement asylum-seeking children and adolescents encounter difficulties establishing friendships and are often subject to social exclusion [53]. These difficulties with peer relationships can contribute to the further development of psychiatric disorders [54].

Children of parents with psychiatric symptoms had more psychiatric symptoms compared to children of parents with no psychiatric symptoms. In addition to the transgenerational processes, it is important to consider that asylum-seeking parents and children might have been exposed to the same traumatic events. Further, with regard to parental assessment of child mental health, parental psychopathology is shown to distort parents’ perceptions of their children’s psychiatric symptoms [55]. The above implies that in order to support mental health of asylum-seeking children, mental health of asylum-seeking parents and family-context needs to be adequately addressed.

It is widely acknowledged that early recognition of children’s psychiatric problems is possible and can support the child’s development and mental health [56, 57]. In addition to developing mental health services targeted at these children and parents, asylum policies need to take into consideration the effect of asylum process stressors on already existing psychiatric symptoms. This applies particularly to young children, considering that asylum policies have been criticized for encompassing children only when they reach the school age or if they are unaccompanied, thus ignoring the youngest of children [58]. Post-arrival factors have been shown to moderate mental health outcomes for asylum seekers [59], suggesting that much can be done after arrival to improve the mental health of children despite their adverse prearrival experiences [60]. Numerous studies have documented that living in a refugee camp, multiple relocations, prolonged asylum processes and a lack of child-friendly immigration procedures are associated with poor mental health outcomes in refugee children [61, 62]. An ecological culturally sensitive approach that takes into account the mental health, living conditions and environmental concerns of children and parents is suggested to increase understanding of psychiatric symptoms of asylum-seeking children as an additive for the Western-based psychiatric approach [41, 63].

## Strengths and limitations

A significant strength of the study is the population-based total sampling design and high participation rate. A further strength is the use of a widely used and validated SDQ instrument for screening psychiatric symptoms among children. It has also been suggested to be suitable for screening recently arrived refugee children [64]. However, some studies have found possible limitations for the use of SDQ with refugee children regarding absent trauma symptom screening and translated subscales that may not measure the same constructs as the UK SDQ [65]. This study relies only on parental assessment of children’s psychiatric symptoms in the absence of other informants shortly after arrival. Both HSCL and PROTECT are broadly used in refugee population, and HSCL is validated in this context, whereas research on psychometric properties of PROTECT is still largely lacking [66, 67]. The measures (SDQ, HSCL-25, PROTECT) were available in languages spoken by the multilingual research assistants, and professional interpreters from accredited companies were used to facilitate administration of the questionnaires in other languages. The possibility for some degree of bias caused by the use of professional interpreters can be regarded as limitation of the study. These remarks on the measures used should be kept in mind when interpreting the results. Nevertheless, all measures behaved in a consistent and predictable way and the results are meaningful in the context of previous literature. The ethnic background and pre-arrival experiences of the asylum-seeking population are influenced by geopolitical conflicts at that time; this affects the generalizability of the results in another time or another place. In this study, multiple ethnically diverse groups with different pre-flight experiences were involved, which is a further strength of the study.

## Summary

This study found that recently arrived accompanied asylum-seeking children had a high prevalence of psychiatric symptoms, and that parents’ anxiety and depression as well as parents’ trauma symptoms were both associated with children’s psychiatric symptoms. Importantly, over half of the under school-aged males had conduct problems above the normal score. The mental health of young accompanied asylum-seeking children should be an important priority in both research and clinical practice. An ecological, culturally sensitive approach that takes into account the mental health, living conditions and environmental concerns of children and parents may increase understanding of the psychiatric symptoms of asylum-seeking children and support their mental health.

### Electronic Supplementary Material

Below is the link to the electronic supplementary material.

Supplementary Material 1
