# Supplementary material for: Psychiatric symptoms and the association with parents’ psychiatric symptoms among recently arrived asylum-seeking children in Finland
Source: Child Psychiatry Hum Dev. 2022 May 19;54(6):1699–709. doi: 10.1007/s10578-022-01371-2 (PMC10582122; doi:10.1007/s10578-022-01371-2)
Supplement: Supplementary file 1 — Supplementary Material 1 [file 10578_2022_1371_MOESM1_ESM.docx]

Online Resource 1

**Supplementary Table 1. Sample characteristics**

|  |  | **Child,**  **2–6 years**  ***n*=93^a^**  ***n* (%)** | **Child,**  **7–12 years *n*=91^a^**  ***n* (%)** |
| --- | --- | --- | --- |
| ***Child*** |  |  |  |
| Age, mean years (range) |  | 4.4 (2.0-6.8) | 9.7 (7.1-12.8) |
| Region of origin | Russia and  Former Soviet Union | 28 (30.1) | 37 (40.7) |
|  | North Africa and Middle East | 53 (57.0) | 32 (35.2) |
|  | Other Africa | 8 (8.6) | 12 (13.2) |
|  | Other countries | <5 | 10 (11.0) |
| Born before 37^th^ gestational week or clearly prematurely |  | 10 (10.9) | 10 (11.4) |
| Learned to walk after 18 months of age or clearly later than peers |  | <5 | <5 |
| First words after 18 months of age or clearly later than peers |  | 14 (15.2) | 10 (11.1) |
| Development and growth not monitored by health care professional |  | 16 (17.4) | 7 (8.0) |
| Present health status poor or quite poor based on the parent's opinion^b^ |  | <5 | <5 |
| ***Mother*** |  | ***n*=73^a^**  ***n* (%)** | ***n*=66**  ***n* (%)** |
| Age, mean years (range) |  | 32.8 (18-46) | 35.5 (27-48) |
| Employed before migration |  | 45 (62.5) | 47 (71.2) |
| Literate | Writing | 69 (94.5) | 60 (90.9) |
|  | Reading | 69 (94.5) | 61 (92.4) |
| ***Father*** |  | ***n*=45**  ***n* (%)** | ***n*=37^a^**  ***n* (%)** |
| Age, mean years (range) |  | 37.0 (26-62) | 38.7 (28-55) |
| Employed before migration |  | 40 (88.9) | 35 (97.2) |
| Literate^c^ | Writing | 44 (98.8) | 37 (100.0) |
|  | Reading | 45 (100.0) | 37 (100.0) |
| ***Family*** |  | ***n*=78**  ***n (%)*** | ***n*=71**  ***n (%)*** |
| Number of children in the family^d^ | 1 | 30 (38.5) | 16 (22.5) |
|  | 2 | 28 (35.9) | 33 (46.5) |
|  | 3 | 13 (16.7) | 16 (22.5) |
|  | ≥4 | 7 (9.0) | 6 (8.5) |

^a^n varies slightly (max n-3) according to measure due to missing information.

^b^Present health status categories: good, quite good, intermediate, quite poor and poor.

^c^Literacy categories: all kinds of text, simple text, read names, words and very simple sentences, and no ability at all. Parents were considered literate in writing or reading if they belonged to the two first-mentioned categories.

^d^Based on the number of children participating in the TERTTU Survey.
